# The protective effect of physical activity on mental health of middle school students at different stages during the COVID-19 outbreak

**DOI:** 10.1038/s41598-024-65599-9

**Published:** 2024-06-26

**Authors:** Ru-bao Dong, Kai-yun Dou, Jiaben Huang, Ronghua Wang

**Affiliations:** 1 Present Address: School of Physical Education, Guizhou Normal University, Guiyang, 550025 China; 2 Graduate School of Adamson University, 0900 Manila, Philippines

**Keywords:** Disease prevention, Public health

## Abstract

This study aims to further understand the changes in physical activity level(PAL) and mental health among adolescents before and after the outbreak of COVID-19 and explore the protective role of physical activity (PA) on the mental health of adolescents during major disasters. A convenient sampling method was used to conduct a cross-sectional survey. The cross-sectional data from 2838 Chinese middle school students (mean age = 14.91 ± 1.71 years, 49.54% female) were used, of which 1,471 and 1,367 were in 2021 and 2022, respectively. The PAL was collected using the Physical Activity Questionnaire for Children (PAQ-CN), mental health status was collected using the Mental Health Inventory of Middle School Students (MMHI-60), sociodemographic information was collected using a self-reported questionnaire. Before and after the outbreak of COVID-19, the PAL of adolescents was 2.36 ± 0.74 and 2.50 ± 0.66, respectively, with a significant difference (*p* < 0.01, 95% CI: 0.09, 0.19). The mental health scores were 1.71 ± 0.60 and 1.86 ± 0.73, respectively, with a significant difference (*p* < 0.01, 95% CI: − 0.20, − 0.10). The detection rates of mental health problems were 27.50% and 35.50%, respectively. The rates of achieving PAL standards were 30.20% and 18.00% among adolescents, while the rates of not achieving PAL standards were 39.60% and 18.00%. PA is a protective factor for the mental health of adolescents during major disasters.

## Introduction

The COVID-19 pandemic has had detrimental effects on both physical activity and the mental health of individuals. Various impacts on personal behavior have resulted from the pandemic^1^. The majority of studies suggest a decline in physical activity among children and adolescents by 10.8–91 min per day during COVID-19^2^. COVID-19 exposure was associated with a higher prevalence of depression symptoms and anxiety symptoms^3^.Individuals affected by COVID-19 may experience a range of psychological issues^4^. Physical activity (PA) is considered a prerequisite for mental well-being^5^. It is a crucial factor in preventing and managing psychological disorders, such as depression and anxiety, as well as promoting mental health^6^. Higher physical activity level(PAL) are associated with lower levels of depressive symptoms, anxiety, and stress, regardless of age, demonstrating a positive correlation between PA and better mental health outcomes^7^.

Although PA has been shown to effectively improve the mental health of both healthy individuals and patients^8,9^. it also plays a role in the prevention and treatment of COVID-19, promoting physical recovery and enhancing psychological well-being^10^. However, mental health issues among children and adolescents have become increasingly serious during the COVID-19 pandemic^11^. Despite this, there is limited research on the relationship between PAL and mental health among adolescents before and after the outbreak of COVID-19. Therefore, this study aims to investigate whether PA serves as a protective factor for the mental health of adolescents during the COVID-19 pandemic. Based on our hypothesis, it is expected that adolescents with higher PAL will exhibit higher levels of mental health, indicating a significant correlation between PAL and mental health levels. Specifically, adolescents with higher PAL are likely to have better mental health and lower rates of psychological issues. The aim of this study is to provide a theoretical basis for implementing physical activity intervention programs for adolescents with lower levels of mental health and to facilitate coordinated mental and physical health promotion. Additionally, it aims to provide convenient intervention methods for safeguarding the mental health of adolescents during major natural disasters.

## Survey participants and methods

### Survey participants

At the end of October 2021, a convenient sampling method was employed to randomly select three senior high schools and six junior high schools as the survey units in Guiyang city, Guizhou Province, China. The participants encompassed male and female students from all grades in the selected schools. The surveyed schools in late October 2022 are the same as those in 2021, but the participants were newly selected through a convenient sampling method. (Note: From January 23, 2020 to January 8, 2023, due to strict epidemic prevention and control measures taken by the Chinese government, COVID-19 only broke out in a few areas of China, while in most areas it was only sporadic. In September 2022, COVID-19 broke out in Guiyang City. Through collaborative efforts from all sectors of society, normal prevention and control measures were resumed from 0:00 on October 5, and offline teaching was fully resumed on October 17).

### Survey tool

The Physical Activity Questionnaire for Children (PAQ-C)^12^ was used to conduct the PAL survey among middle school students. This questionnaire was chosen primarily because "physical activities during recess" are commonly arranged in Chinese primary and secondary schools. The PAQ-C is known for its capability to sensitively differentiate between genders and age groups. It does not require a detailed recollection of exercise time or intensity, and its questions are clear, easy to understand, and straightforward to complete. This helps minimize recall bias^13^. Additionally, the retest reliability of PAQ-C ɑ = 0.81, criterion validity is 0.48^13^; the internal consistency reliability coefficient (Cronbach's alpha) ɑ = 0.821, KMO (Kaiser–Meyer–Olkin) is 0.878^14^. PAQ-C adopts a Likert 5-point scoring system, with a total of 10 items. The 10th item is a screening question to understand whether the subject has been sick (such as a bad cold, etc.) or other exceptional case that have affected their normal PA in the past week. Therefore, special groups of subjects are excluded from the sample, and this item is not included in the final total physical activity score. Item 1 (Spare time activity) : Take the mean of all activities (“no” activity being a 1, “7 times or more” being a 5) on the activity checklist to form a composite score for item 1. Items 2 to 8 (PE, recess, lunch, right after school, evening, weekends, and describes you best): The answers for each item start from the lowest activity response and progress to the highest activity response. Simply use the reported value that is checked off for each item (the lowest activity response being a 1 and the highest activity response being a 5). Item 9: Take the mean of all days of the week (“none” being a 1, “very often” being a 5) to form a composite score for item 9. The total score of PAQ-C is the average score of items 1–9, and the lower the score, the lower the level of physical activity^12^.

MMHI-60 is a scale developed by Chinese scholars specifically for evaluating the mental health of Chinese middle school students. The scale consists of 60 items and adopts a Likert 5-level rating system. The survey content includes the overall mental health status and 10 factors of obsessive–compulsive symptoms, bigotry, hostility, interpersonal tension and sensitivity, depression, anxiety, learning pressure, maladaptation, emotional instability, and psychological imbalance among middle school students; The scores of each factor are composed of the average scores of each item in each subscale, and the average scores of all items constitute the total average score (measuring overall mental health status, abbreviated as psychological status); A total average score and an average score of ≥ 2 for each factor indicate mild symptoms of mental health, and the higher the score, the more serious the psychological problem. This questionnaire has good reliability and validity, retest reliability = 0.72 ~ 0.91, structural validity = 0.47 ~ 0.76^15^, but each subscale cannot be used separately. The internal consistency reliability coefficient (Cronbach's alpha) ɑ = 0.970, and its KMO(Kaiser–Meyer–Olkin) was 0.980 in the study.

The self-administered questionnaire includes basic information about the survey participants, such as gender, grade, and age.

### Sampling basis

The formula for sample size calculation is as follows: N = Z^2^ × (P × (1 − P))/E^2^^16^, where N represents the sample size, Z is the statistical value of the 95% confidence interval (Z = 1.96), E is equal to 0.05, and P is 0.5. Hence, N ≈ 385. Due to significant differences in cognitive abilities and academic pressures between junior high school students and senior high school students, this study considered them as two separate groups and conducted separate sampling. The sample sizes in this study exceeded the minimum requirements.

### Questionnaire survey

The study was conducted after obtaining approval from the Academic Ethics Committee of the School of Physical Education at Guizhou Normal University, all methods were performed in the study in accordance with the WMA DoH 1964–2014. Physical education teachers from various schools verbally recruited participants and distributed informed consent forms. One week later, the consent forms, signed by the parents, were collected to allow the participants to take part in the questionnaire survey. The surveys were conducted on rainy days in October 2021 (during the period of normal prevention and control measures) and late October 2022 (immediately after the lifting of COVID-19 lockdown). The investigators gathered the respondents in classrooms and distributed, completed, and collected the questionnaires. In 2021, the sample size of junior and senior high school students was 891 and 598, respectively; and in 2022, the sample size of junior and senior high school students was 580 and 769, respectively. The sample size of both junior and senior high school students in this study exceeded the minimum sample size required for the social survey, indicating that the sample was well representative (see Table 1).

**Table 1:** Distribution of respondents.

Year	Junior high school students			Senior high school students			Sex		Average age (M ± SD)	Total
	7	8	9	10	11	12	Male	Female		
2021	328	299	264	222	216	142	741	730	14.92 ± 1.76	1471
2022	170	236	192	284	280	205	691	676	14.90 ± 1.66	1367
subtotal	498	535	456	506	496	347	1432	1406	14.91 ± 1.71	2838

### Data statistical method

Statistical analysis of the data was conducted in IBM SPSS Statistics 26.0. Pearson correlation was used to examine the relationship, one-way ANOVA was used to compare the differences between PAL and mental health among middle school students; and OR (odds ratios) was used to further understand the differences in the detection rate of psychological problems between middle school students who achieve the recommended PAL and those who do not at different stages during COVID-19. The significance level was set at* p* < 0.05.

## Results

PA plays a role in the prevention and treatment of COVID-19, promoting physical recovery and enhancing psychological well-being^10^. Therefore, adolescent PAL was the independent variable and MH was the dependent variable in the study.

### Relationship and comparison between PAL and MH in adolescents.

Compared to before the outbreak of COVID-19, the PAL among adolescents has decreased, while their psychological state (as measured by the average scores) and the scores for its ten factors have increased. Specifically, the average scores for learning pressure (academic pressure) and emotional instability have surpassed the threshold for a healthy level by two points. The mean scores for the factors of obsessive–compulsive symptoms and anxiety have also approached the threshold. Therefore, it can be concluded that after the COVID-19 outbreak, there was a significant decline in both PAL and mental health among adolescents.

In terms of the learning stages of high school and middle school students, the mental health and various psychological factors, including PAL and mental state, significantly decreased after COVID-19. Additionally, only the average scores of four factors, namely, mental imbalance, bigotry, hostility, and interpersonal relationships, did not reach the critical threshold after the outbreak. On the other hand, among middle school students, only PAL showed a significant decrease, while there were no significant changes in mental health levels. Furthermore, the levels of bigotry and interpersonal relationships were higher than before the COVID-19 outbreak among middle school students (Table 2).

**Table 2:** Comparison of PAL and MH among junior and senior high school students between pre- and post- COVID-19.

	year	All samples							Senior high school students							Junior high school students						
		N	%	Mean	Std.D	Std.E	t	p	N	%	Mean	Std.D	Std.E	t	p	N	%	Mean	Std.D	Std.E	t	p
PAL	2021	1471	100	2.50	0.66	0.02	5.30	0.000	580	39.43	2.27	0.62	0.03	2.00	0.045	891	60.57	2.65	0.65	0.02	2.19	0.029
	2022	1367	100	2.36	0.74	0.02			769	56.25	2.20	0.73	0.03			598	43.75	2.57	0.69	0.03		
Mental status	2021	1471	100	1.71	0.60	0.02	− 5.82	0.000	580	39.43	1.82	0.63	0.03	− 5.40	0.000	891	60.57	1.64	0.58	0.02	− 0.12	0.902
	2022	1367	100	1.86	0.73	0.02			769	56.25	2.02	0.76	0.03			598	43.75	1.65	0.62	0.03		
Psychological imbalance	2021	1471	100	1.54	0.61	0.02	− 4.33	0.000	580	39.43	1.63	0.64	0.03	− 3.84	0.000	891	60.57	1.47	0.58	0.02	0.08	0.935
	2022	1367	100	1.64	0.72	0.02			769	56.25	1.78	0.79	0.03			598	43.75	1.47	0.59	0.02		
Anxiety	2021	1471	100	1.73	0.81	0.02	− 6.22	0.000	580	39.43	1.83	0.84	0.03	− 5.83	0.000	891	60.57	1.67	0.78	0.03	− 0.89	0.376
	2022	1367	100	1.94	0.97	0.03			769	56.25	2.13	1.01	0.04			598	43.75	1.71	0.87	0.04		
Academic pressure	2021	1471	100	1.79	0.80	0.02	− 6.81	0.000	580	39.43	1.95	0.85	0.04	− 6.28	0.000	891	60.57	1.68	0.75	0.03	− 0.22	0.824
	2022	1367	100	2.01	0.96	0.03			769	56.25	2.26	1.02	0.04			598	43.75	1.69	0.79	0.03		
Emotional instability	2021	1471	100	1.88	0.75	0.02	− 5.94	0.000	580	39.43	2.01	0.75	0.03	− 5.48	0.000	891	60.57	1.80	0.74	0.02	− 0.48	0.629
	2022	1367	100	2.06	0.87	0.02			769	56.25	2.25	0.88	0.03			598	43.75	1.82	0.79	0.03		
Bigotry	2021	1471	100	1.68	0.68	0.02	− 3.90	0.000	580	39.43	1.77	0.71	0.03	− 3.70	0.000	891	60.57	1.61	0.66	0.02	0.46	0.645
	2022	1367	100	1.78	0.77	0.02			769	56.25	1.93	0.82	0.03			598	43.75	1.60	0.65	0.03		
Hostility	2021	1471	100	1.57	0.67	0.02	− 3.94	0.000	580	39.43	1.64	0.68	0.03	− 3.03	0.002	891	60.57	1.52	0.67	0.02	− 1.18	0.238
	2022	1367	100	1.68	0.81	0.02			769	56.25	1.77	0.84	0.03			598	43.75	1.56	0.75	0.03		
Interpersonal relationship	2021	1471	100	1.67	0.69	0.02	− 4.59	0.000	580	39.43	1.77	0.72	0.03	− 4.39	0.000	891	60.57	1.61	0.66	0.02	0.13	0.898
	2022	1367	100	1.80	0.82	0.02			769	56.25	1.96	0.86	0.03			598	43.75	1.60	0.73	0.03		
Depression	2021	1471	100	1.71	0.79	0.02	− 4.70	0.000	580	39.43	1.81	0.79	0.03	− 4.34	0.000	891	60.57	1.65	0.79	0.03	− 0.32	0.746
	2022	1367	100	1.86	0.92	0.03			769	56.25	2.02	0.98	0.04			598	43.75	1.66	0.80	0.03		
Obsessive- compulsive	2021	1471	100	1.90	0.66	0.02	− 2.96	0.003	580	39.43	1.95	0.65	0.03	− 4.12	0.000	891	60.57	1.87	0.66	0.02	1.69	0.092
	2022	1367	100	1.98	0.72	0.02			769	56.25	2.11	0.74	0.03			598	43.75	1.81	0.65	0.03		
Maladaptation	2021	1471	100	1.64	0.64	0.02	− 6.65	0.000	580	39.43	1.81	0.68	0.03	− 5.30	0.000	891	60.57	1.54	0.60	0.02	− 0.84	0.400
	2022	1367	100	1.83	0.80	0.02			769	56.25	2.03	0.84	0.03			598	43.75	1.56	0.64	0.03		

Std.D = Std.Deviation, Std.E = Std.Error.

There is a significant correlation between PAL and the psychological state as well as the scores of various factors in adolescents. The absolute value of the correlation coefficient ɑ between adolescent PAL and mental status and its 10 psychological factors, pre-COVID-19: ɑ = 0.132 ~ 0.272 for all samples (all), ɑ = 0.142 ~ 0.303 for Junior high school students (Junior), ɑ = 0.079 ~ 0.156 for Senior high school students(Senior); post-COVID-19, ɑ = 0.178 ~ 0.336 for all samples, ɑ = 0.135 ~ 0.292 for Junior, ɑ = 0.131 ~ 0.294 for Senior.

However, following the outbreak of COVID-19, the absolute values of the correlation coefficients between the psychological state and its 10 factors and PAL have increased, indicating a stronger correlation. Specifically, for middle school and high school students, except for the reduced absolute values of the correlation coefficients between middle school students' psychological imbalance and obsessive–compulsive symptoms factors and PAL after the outbreak of COVID-19, the absolute values of correlation coefficients between all other psychological factors and PAL have increased. Among them, the correlation between high school students' hostility factor and PAL changed from non-significant to highly significant after the COVID-19 outbreak. Furthermore, when comparing the correlation coefficients between PAL and the factors related to mental health in middle school and high school students in the same year, except for the higher absolute value of the correlation coefficient between high school students' maladjustment and PAL after the outbreak of COVID-19, the absolute values of the correlation coefficients between all other psychological factors and PAL were lower in high school students than in middle school students.

### Comparison of PAL and MH among male and female in junior and senior high school students

Compared to the period pre-COVID-19, there was a significant decrease in the levels of PAL and overall mental health for male high school students during the outbreak, with the exception of a non-significant decrease in physical health. Similarly, there was a significant decrease in all mental health levels for female high school students, except for a non-significant decrease in PAL and mental imbalance factor health levels.

There were no significant changes in PAL and mental health levels for both male and female middle school students before and after the COVID-19 outbreak. However, male students experienced a decrease in PAL and academic pressure levels, while their mental state and overall mental health levels showed improvement after the outbreak. On the other hand, female middle school students saw an improvement in their obsessive–compulsive symptoms and interpersonal relationship factors but experienced a slight decline in PAL, mental state, and other psychological health levels (Table 3).

**Table 3:** Comparison of PAL and MH of junior and senior high school students of different genders between pre- and post- COVID-19.

	year	Male of senior high school students							Female of senior high school students							Male of junior high school students							Female of junior high school students						
		N	%	Mean	Std.D	Std.E	t	p	N	%	Mean	Std.D	Std.E	t	p	N	%	Mean	Std.D	Std.E	t	p	N	%	Mean	Std.D	Std.E	t	p
PAL	2021	306	20.80	2.47	0.63	0.04	2.08	0.038	274	18.63	2.04	0.54	0.03	0.59	0.556	435	29.57	2.82	0.68	0.03	1.76	0.080	456	31.00	2.48	0.56	0.03	1.45	0.149
	2022	399	29.19	2.36	0.77	0.04			370	27.07	2.01	0.63	0.03			292	21.36	2.73	0.73	0.04			306	22.38	2.42	0.62	0.04		
Mental status	2021	306	20.80	1.81	0.63	0.04	− 3.65	0.000	274	18.63	1.83	0.63	0.04	− 3.99	0.000	434	29.57	1.59	0.55	0.03	0.61	0.541	456	31.00	1.70	0.60	0.03	− 0.71	0.480
	2022	399	29.19	2.01	0.78	0.04			370	27.07	2.04	0.73	0.04			292	21.36	1.56	0.58	0.03			306	22.38	1.73	0.65	0.04		
Psychological imbalance	2021	306	20.80	1.63	0.65	0.04	− 3.60	0.000	274	18.63	1.63	0.64	0.04	− 1.75	0.080	435	29.57	1.46	0.55	0.03	0.24	0.814	456	31.00	1.49	0.61	0.03	− 0.10	0.918
	2022	399	29.19	1.83	0.83	0.04			370	27.07	1.72	0.74	0.04			292	21.36	1.45	0.58	0.03			306	22.38	1.49	0.60	0.03		
Anxiety	2021	306	20.80	1.80	0.85	0.05	− 4.17	0.000	274	18.63	1.87	0.83	0.05	− 4.06	0.000	435	29.57	1.58	0.72	0.03	0.31	0.757	456	31.00	1.75	0.82	0.04	− 1.38	0.170
	2022	399	29.19	2.10	1.02	0.05			370	27.07	2.16	1.00	0.05			292	21.36	1.56	0.76	0.04			306	22.38	1.84	0.95	0.05		
Academic pressure	2021	306	20.80	1.90	0.84	0.05	− 3.95	0.000	274	18.63	1.99	0.85	0.05	− 4.93	0.000	435	29.57	1.59	0.72	0.03	-0.04	0.965	456	31.00	1.77	0.77	0.04	− 0.27	0.790
	2022	399	29.19	2.18	0.99	0.05			370	27.07	2.36	1.03	0.05			292	21.36	1.59	0.74	0.04			306	22.38	1.79	0.81	0.05		
Emotional instability	2021	306	20.80	1.99	0.73	0.04	− 3.74	0.000	274	18.63	2.03	0.77	0.05	− 4.00	0.000	435	29.57	1.72	0.70	0.03	0.00	0.998	456	31.00	1.87	0.76	0.04	− 0.65	0.519
	2022	399	29.19	2.22	0.90	0.04			370	27.07	2.29	0.86	0.04			292	21.36	1.72	0.73	0.04			306	22.38	1.91	0.83	0.05		
Bigotry	2021	306	20.80	1.81	0.74	0.04	− 2.28	0.023	274	18.63	1.74	0.67	0.04	− 3.03	0.003	435	29.57	1.57	0.63	0.03	0.90	0.368	456	31.00	1.65	0.68	0.03	− 0.19	0.846
	2022	399	29.19	1.94	0.86	0.04			370	27.07	1.91	0.77	0.04			292	21.36	1.53	0.63	0.04			306	22.38	1.66	0.67	0.04		
Hostility	2021	306	20.80	1.66	0.66	0.04	− 1.83	0.068	274	18.63	1.62	0.70	0.04	− 2.47	0.014	435	29.57	1.50	0.65	0.03	0.30	0.762	456	31.00	1.54	0.68	0.03	− 1.82	0.069
	2022	399	29.19	1.76	0.85	0.04			370	27.07	1.77	0.83	0.04			292	21.36	1.48	0.68	0.04			306	22.38	1.64	0.81	0.05		
Interpersonal relationship	2021	306	20.80	1.76	0.69	0.04	− 3.79	0.000	274	18.63	1.78	0.76	0.05	− 2.36	0.019	435	29.57	1.54	0.63	0.03	0.16	0.870	456	31.00	1.67	0.68	0.03	0.03	0.980
	2022	399	29.19	1.98	0.89	0.04			370	27.07	1.93	0.83	0.04			292	21.36	1.53	0.70	0.04			306	22.38	1.67	0.75	0.04		
Depression	2021	306	20.80	1.77	0.77	0.04	− 2.78	0.006	274	18.63	1.86	0.82	0.05	− 3.33	0.001	435	29.57	1.54	0.73	0.03	0.34	0.735	456	31.00	1.74	0.83	0.04	− 0.70	0.486
	2022	399	29.19	1.95	0.92	0.05			370	27.07	2.11	1.03	0.05			292	21.36	1.53	0.68	0.04			306	22.38	1.79	0.89	0.05		
Obsessive- compulsive	2021	306	20.80	1.94	0.66	0.04	− 2.69	0.007	274	18.63	1.96	0.64	0.04	− 3.14	0.002	435	29.57	1.82	0.66	0.03	1.82	0.069	456	31.00	1.91	0.65	0.03	0.60	0.547
	2022	399	29.19	2.09	0.77	0.04			370	27.07	2.13	0.72	0.04			292	21.36	1.73	0.62	0.04			306	22.38	1.88	0.68	0.04		
Maladaptation	2021	306	20.80	1.84	0.71	0.04	− 3.08	0.002	274	18.63	1.78	0.64	0.04	− 4.54	0.000	435	29.57	1.53	0.61	0.03	0.58	0.562	456	31.00	1.54	0.58	0.03	− 1.76	0.079
	2022	399	29.19	2.02	0.88	0.04			370	27.07	2.04	0.80	0.04			292	21.36	1.51	0.62	0.04			306	22.38	1.62	0.65	0.04		

### Comparison of the detection rates of MH problems among male and female

The detection rate (DR) of health problem in adolescent mental status and its 10 psychological factors, pre-COVID-19: DR = 21.9% ~ 42.0% for all, DR = 18.6% ~ 36.8% for male of Junior, DR = 18.4% ~ 43.2% for female of Junior, DR = 29.1% ~ 48.0% for male of Senior, and DR = 24.8% ~ 46.7% for female of Senior; post-COVID-19, DR = 26.2% ~ 49.5% for all, DR = 14.0% ~ 31.2% for male of Junior, DR = 18.6% ~ 38.9% for female of Junior, DR = 34.8% ~ 59.1% for male of Senior, and DR = 25.9% ~ 62.4% for female of Senior.

Overall, there has been an increase in the detection rate of mental health issues among adolescents after the outbreak of COVID-19 compared to before the outbreak. For middle school boys, there has been a slight increase in the detection rate of emotional instability, while the detection rates of all other mental health issues have decreased. Among middle school girls, the detection rates of academic pressure, bigotry, depression, and obsessive–compulsive symptoms have also decreased. However, the detection rates of all mental health issues have increased for both male and female high school students. Before and after the COVID-19 outbreak, the detection rates of all mental health issues among middle school students were lower than those among high school students. Before the outbreak, the detection rates of mental imbalance, hostility, and maladjustment were higher among middle school boys compared to middle school girls, and the detection rates of mental imbalance, emotional instability, bigotry, hostility, obsessive–compulsive symptoms, and maladjustment were higher among high school boys compared to high school girls. After the outbreak, all mental health issues had lower detection rates among middle school boys compared to middle school girls, while among high school boys, only the detection rates of mental state, mental imbalance, bigotry, hostility, and interpersonal relationship issues were higher compared to high school girls (the detection rates of emotional instability, obsessive–compulsive symptoms, and maladjustment became lower compared to high school girls, but the detection rates of mental state and interpersonal relationship issues became higher compared to high school girls).

### Comparison between adolescent PAL and detection rate of psychological issues

According to the criteria for classifying PAL, adolescent PAL can be divided into two categories: meeting the standards and not meeting the standards. Specifically, PAL ≤ 3 indicates not meeting the standards, while PAL > 3 indicates meeting the standards^17^.

The detection rate of problems for mental status and 10 psychological factors in adolescents who did not meet the recommended PAL, for all samples, pre-COVID-19, DR = 23.1% ~ 43.8%, post-COVID-19, DR = 28.1% ~ 54.9%, with an increase in DR of 5.0% ~ 11.1%; Junior, pre- COVID-19, DR = 20.0% ~ 43.3%, post-COVID-19, DR = 18.6% ~ 38.4%, DR increase range is -4.9% ~ 3.6%, there are four DR of psychological problems decreases after the outbreak of COVID-19; Senior, pre-COVID-19, DR = 26.9% ~ 48.6%, post-COVID-19, DR = 34.4% ~ 63.4%, with an increase in DR of 6.9% ~ 16.8%. The DR of problems in adolescents who have reached the recommended PAL for their mental status and 10 psychological factors. For all samples, pre-COVID-19, DR = 17.7% ~ 35.3%, and post- COVID-19, DR = 15.3% ~ 34.5%, with an increase in detection rate of -6.5% ~ 1.8%. Only 4 DR of psychological problems had an increase. Junior, pre-COVID-19, DR = 14.6% ~ 31.6%, post-COVID-19, DR = 7.0% ~ 24.7%, DR increase range is -10.4% ~ -4.5%, all psychological problems detection rate of junior high school students decreases after the outbreak of COVID-19. Senior, pre-COVID-19, DR = 28.6% ~ 48.6%, post-COVID-19, DR = 28.2% ~ 49.5%, DR increase range from − 11.8% to 12.7%, only 2 psychological problems have a decrease in DR. Following the outbreak of COVID-19, all aspects of psychological problems among the overall sample who were not labeled as PAL increased compared to before the outbreak. In contrast, PAL-compliant individuals experienced an increase in the detection rates of three factors: psychological imbalance, academic pressure, and emotional instability. However, the detection rates of the other seven psychological factors decreased, while the detection rate for mental states remained unchanged. For those who did not meet the PAL standards, the detection rates for all psychological issues increased.

After the outbreak of COVID-19 among middle school students, there was an increase in the detection rate of six factors related to psychological state and maladjustment among those who did not meet PAL standards: maladaptation, depression, interpersonal tension and sensitivity, hostility, emotional instability, and anxiety. Conversely, among PAL-compliant students, the detection rate of all psychological issues decreased. Among high school students, apart from a decrease in the detection rate of depression and maladjustment among PAL-compliant students, the detection rate of all other psychological issues, regardless of PAL compliance, increased after the COVID-19 outbreak. Additionally, in terms of the detection rate of psychological issues during the same period, PAL-compliant students had a lower detection rate compared to PAL non-compliant students (except for the four factors of psychological imbalance, hostility, obsessive–compulsive symptoms, and maladjustment among high school students before the COVID-19 outbreak). Overall, there was a higher detection rate of psychological issues among adolescents after the COVID-19 outbreak, with a particularly pronounced detection rate among PAL non-compliant students.

Compared to the pre-COVID-19, there has been a slight increase in the DR of emotional instability issues among male middle school students who did not meet the PAL standards. However, the DRs of all other psychological problems have decreased for male middle school students. Among female middle school students, the DR of obsessive–compulsive symptoms has decreased for those who did not meet the PAL standards, while the DRs of all other psychological problems have increased. For male high school students, there has been a slight decrease in the DRs of anxiety, bigotry, interpersonal tension and sensitivity, depression, and maladjustment—these five psychological factors—among those who met the PAL standards. However, the DRs of all other psychological problems have increased. Among male high school students who did not meet the PAL standards, the DRs of all psychological problems have increased. Similarly, among female high school students who did not meet the PAL standards, the DRs of all psychological problems have increased, while among those who met the PAL standards, there has been a slight decrease in the DR of emotional instability factors, no change in the DR of obsessive–compulsive symptoms factors, and an increase in the DRs of all other psychological problems. Additionally, considering the DRs of psychological problems, it can be observed that the impact of the COVID-19 outbreak on the mental health of high school students is greater than that of middle school students. Within the same stage of education, the impact on female students is greater than that on male students. Furthermore, the impact is greater on those who did not meet the PAL standards compared to those who met the standards.

A comparison using OR reveals that, apart from the significant beneficial effects of the COVID-19 outbreak on the hostility, interpersonal tension, and maladaptation of male middle school students who meet the PAL standards, there were no significant positive effects on the DRs of other psychological issues in this group. For middle school girls, the outbreak has a significant positive influence on the DR of maladjustment and hostility factors in those who do not meet the PAL standard but has no significant impact on other psychological issues. However, for girls who meet the PAL standard, the outbreak has a significant positive influence on anxiety, academic pressure, and hostility factors but no significant impact on the DR of other factors. Regarding high school boys, the COVID-19 outbreak has a significant negative impact on those who do not meet the PAL standard (excluding bigotry and hostility factors) but no significant impact on those who meet the PAL standard. On the other hand, for high school girls, the outbreak has a significant negative impact on the DR of psychological imbalances and bigotry, excluding hostility factors, in those who do not meet the PAL standard. However, it does not have a significant negative impact on those who meet the PAL standard (Table 4).

**Table 4:** Comparison of the prevalence and Odds Ratio of Psychological Problems between pre- and post-COVID-19 (N_2021_ = 1471, N_2022_ = 1367).

PAL	Factors	Male of junior (N_MJ_ = 727)						Female of junior (N_FJ_ = 762)						Male of senior (N_MS_ = 705)						Female of senior (N_FS_ = 644)					
		2021 (%)	2022 (%)	Subtotal (%)	Val	95% CI		2021 (%)	2022 (%)	Subtotal (%)	Val	95% CI		2021 (%)	2022 (%)	Subtotal (%)	Val	95% CI		2021	2022	subtotal	Val	95% CI	
						Lo	Up					Lo	Up					Lo	Up					Lo	Up
Not meet the standard	Mental status	25.20	21.90	23.80	0.83	0.54	1.30	27.50	31.20	29.00	1.20	0.84	1.70	33.90	49.70	42.80	1.93	1.37	2.71	35.90	45.90	41.60	1.52	1.09	2.11
	Psychological imbalance	20.30	16.00	18.50	0.75	0.46	1.23	19.80	20.60	20.10	1.05	0.70	1.55	29.00	39.50	35.00	1.60	1.12	2.27	24.80	29.50	27.50	1.27	0.88	1.83
	Anxiety	30.10	26.70	28.70	0.85	0.56	1.29	33.90	39.90	36.30	1.30	0.93	1.81	37.10	54.00	46.70	1.99	1.42	2.79	38.90	53.20	47.00	1.78	1.29	2.47
	Academic pressure	32.30	27.30	30.20	0.79	0.52	1.19	36.50	38.70	37.40	1.10	0.79	1.53	41.10	57.70	50.50	1.95	1.40	2.73	45.40	62.30	55.00	1.98	1.43	2.75
	Emotional instability	36.10	39.00	37.30	1.13	0.77	1.67	42.90	44.30	43.40	1.06	0.77	1.46	50.00	61.70	56.60	1.61	1.15	2.25	47.30	64.90	57.30	2.06	1.48	2.86
	Bigotry	29.30	25.70	27.80	0.83	0.55	1.27	28.30	28.50	28.40	1.01	0.71	1.43	36.70	44.40	41.10	1.38	0.98	1.94	35.10	41.80	38.90	1.33	0.95	1.85
	Hostility	25.20	21.40	23.60	0.81	0.52	1.26	22.20	31.20	25.80	1.59	1.11	2.28	31.00	36.10	33.90	1.26	0.88	1.78	26.00	34.50	30.80	1.50	1.05	2.14
	Interpersonal relationship	28.20	27.30	27.80	0.96	0.63	1.45	30.20	32.80	31.20	1.13	0.80	1.59	35.10	47.50	42.10	1.68	1.19	2.36	34.70	44.20	40.10	1.49	1.07	2.07
	Depression	27.80	26.70	27.40	0.95	0.62	1.44	36.00	37.20	36.50	1.05	0.76	1.46	33.90	45.40	40.40	1.62	1.15	2.28	40.10	50.00	45.70	1.50	1.08	2.07
	Obsessive–compulsive	40.20	33.70	37.50	0.76	0.51	1.12	45.50	41.90	44.10	0.86	0.63	1.19	44.80	55.20	50.70	1.52	1.09	2.13	44.30	57.30	51.70	1.69	1.22	2.34
	Maladaptation	27.80	23.00	25.80	0.78	0.50	1.20	23.30	31.20	26.50	1.50	1.05	2.14	38.70	50.60	45.50	1.62	1.16	2.27	36.30	50.30	44.20	1.78	1.28	2.47
Meet the standard	Mental status	14.80	9.50	12.80	0.61	0.28	1.32	15.40	5.70	11.50	0.33	0.09	1.23	31.00	32.00	31.60	1.05	0.50	2.19	16.70	35.70	30.00	2.78	0.51	15.26
	Psychological imbalance	16.00	10.50	13.90	0.62	0.29	1.30	11.50	9.40	10.70	0.80	0.25	2.53	29.30	30.70	30.10	1.07	0.51	2.26	25.00	28.60	27.50	1.20	0.26	5.61
	Anxiety	18.90	11.40	16.10	0.55	0.27	1.13	21.80	7.50	16.00	0.29	0.09	0.93	36.20	36.00	36.10	0.99	0.49	2.02	33.30	39.30	37.50	1.29	0.31	5.35
	Academic pressure	20.70	14.30	18.20	0.64	0.33	1.24	26.90	11.30	20.60	0.35	0.13	0.93	32.80	42.70	38.30	1.53	0.75	3.12	33.30	53.60	47.50	2.31	0.56	9.47
	Emotional instability	20.10	17.10	19.00	0.82	0.44	1.55	21.80	11.30	17.60	0.46	0.17	1.25	39.70	48.00	44.40	1.41	0.70	2.81	33.30	32.10	32.50	0.95	0.23	3.99
	Bigotry	18.30	10.50	15.30	0.52	0.25	1.09	14.10	5.70	10.70	0.37	0.10	1.38	31.00	30.70	30.80	0.98	0.47	2.06	33.30	39.30	37.50	1.29	0.31	5.35
	Hostility	16.60	7.60	13.10	0.42	0.18	0.95	19.20	5.70	13.70	0.25	0.07	0.92	29.30	29.30	29.30	1.00	0.47	2.13	25.00	28.60	27.50	1.20	0.26	5.61
	Interpersonal relationship	17.80	8.60	14.20	0.43	0.20	0.96	17.90	9.40	14.50	0.48	0.16	1.41	32.80	34.70	33.80	1.09	0.53	2.25	33.30	35.70	35.00	1.11	0.27	4.63
	Depression	16.00	11.40	14.20	0.68	0.33	1.41	17.90	5.70	13.00	0.27	0.08	1.01	36.20	28.00	31.60	0.69	0.33	1.43	25.00	35.70	32.50	1.67	0.37	7.61
	Obsessive–compulsive	31.40	24.80	28.80	0.72	0.42	1.25	32.10	24.50	29.00	0.69	0.31	1.51	48.30	49.30	48.90	1.04	0.53	2.07	50.00	50.00	50.00	1.00	0.26	3.87
	Maladaptation	18.30	6.70	13.90	0.32	0.14	0.75	12.80	7.50	10.70	0.56	0.16	1.87	44.80	32.00	37.60	0.58	0.29	1.18	16.70	17.90	17.50	1.09	0.18	6.58

## Discussion

The PAL of teenagers ranged from 2.36 to 2.50, which is lower than the levels reported in previous studies before the COVID-19 pandemic^14,18^. Consistent with studies conducted before the pandemic^19–21^, boys had higher PAL than girls, and PAL was higher among middle school students compared to high school students. Similar trends in PAL changes were observed among younger middle school students^22^ and female high school students^2,22^ during the COVID-19 period. The outbreak of COVID-19 resulted in a significant decrease in PAL among teenagers, primarily due to the stringent preventive measures implemented by the Chinese government to contain the spread of the virus. These measures had a negative impact on the physical activities of teenagers as they restricted personal mobility, daily routines, and social interactions^23,24^, aligning with findings that suggest a sharp decline in physical activity time among children and adolescents during the COVID-19 pandemic^25^. However, it is noteworthy that only the PAL of high school boys exhibited a significant decrease, which differed from the findings of related research^2^. This divergence may be attributed to the relatively higher PAL of high school boys under normal circumstances when compared to girls.

The overall prevalence rate of psychological problems among adolescents ranges from 27.50% to 35.50%, which is lower than the reported "nearly 40.4% of adolescents showing a tendency towards psychological problems"^26^. The detection rate of depression problems ranges from 31.50% to 37.20%, which is higher than the reported "general incidence rate of depression symptoms among Chinese children and adolescents at 15.4%"^27^ but lower than 43.7%^28^. The detection rate of anxiety problems ranges from 32.40% to 41.10%, which is similar to the 37.4% anxiety symptoms reported among high school students, where high school is considered a risk factor for depression and anxiety symptoms^28^. After the outbreak of COVID-19, the prevalence rate of psychological problems among adolescents has increased. This is attributed to the disruption of normal life due to government-imposed lockdowns or stay-at-home orders, which significantly impact the mental health of the affected individuals^29,30^, It is also related to the concerns of adolescents regarding isolation and the impact on their education. The research was conducted in October 2021 and 2022, during which the surveyed locations were in different stages of pandemic control, either "normal prevention and control" (with the requirement of a "health code" for travel but maintaining social distancing) or "emergency/static management" (residents were prohibited from leaving their homes except for nucleic acid testing or city lockdown). The prevalence rate of psychological problems among adolescents during the COVID-19 period falls within the normal range^31^, possibly due to various channels, such as hotlines, online consultations, and outpatient services, through which the Chinese government provides mental health services^32^.

During the period of regular prevention and control, the PAL of middle school students was found to be higher than that of high school students, and the PAL of male students was higher than that of female students. Additionally, there was a gradual increase in the detection rates of psychological problems, anxiety, and depression among middle school boys, middle school girls, high school boys, and high school girls. Following the outbreak of COVID-19, the detection rate of psychological problems among high school boys was slightly higher than that of high school girls, possibly due to a significant decrease in their PAL. The detection rate of psychological problems among girls was higher than that of boys, similar to the findings of previous studies^28,33^. The relatively high detection rate of psychological problems among high school students is associated with the greater pressure of academic demands on students^28^.

The outbreak of COVID-19 has had no significant impact on the prevalence of psychological issues among middle school students, except for a significant decrease in maladaptation rates among male students. However, it has had a significant negative effect on the prevalence of most psychological factors among high school students, with a slightly greater negative impact on female students compared to male students. Analyzing the prevalence of psychological issues among middle school and high school students who meet or do not meet PA standards reveals that PA is not only a protective factor for adolescent mental health but also has a more pronounced protective effect on male and younger adolescents. It seems that the relationship between PA and mental health is not entirely unrelated to age, contrary to what is suggested by the previous study^7^.

The absolute values of the correlation coefficients between PAL and psychological factors scores among adolescents have generally increased following the outbreak of COVID-19. This not only implies that "regular physical activity has positive effects on various aspects of health"^34^ and that "the mental and physical health of adolescents is interconnected"^35^, but also suggests that active PAL serves as a protective factor for the mental health of adolescents during significant natural disasters. Additionally, there is a trend indicating that lower grade levels (or younger age) are associated with a stronger protective effect of PAL on mental health.

China's successful containment of the COVID-19 virus is largely attributed to the rapid implementation of quarantine measures; And this measure has also been imitated by other countries^35^. However, these prevention and control measures limit individual mobility, daily activities^36^, and social interaction^23^. During COVID-19, physical distancing/isolation measures and the resulting stressors may have negative effects on mental health such as anxiety, depression, and behavioral problems^37^. During the COVID-19 pandemic, higher levels of physical activity have a certain protective effect on the mental health of adolescents. Therefore, in the event of a similar COVID-19 disaster and restrictions on social activities, it is necessary to provide physical activity opportunities for adolescents and create conditions to encourage them to actively participate in various physical activities. In this way, it can effectively alleviate the adverse effects of major disasters and social prevention and control on the mental health of adolescents, as well as enhance their physical health level, especially for younger male adolescents.

### Research insufficiency and limitations

Due to the impact of COVID-19 prevention and control policies, convenience sampling was adopted in the study. The survey subjects were all from Guiyang City and were not from the same group, so the representativeness of the sample and the accuracy of vertical comparison may be limited; The data comes from subjective surveys, and the accuracy of the data may be influenced by factors such as survey bias, social expectations, and recall bias; At the same time, differences in the level of socio-economic development and culture among different regions, especially the differences in epidemic prevention policies adopted during the COVID-19 pandemic, may also have different impacts on the PA and mental health of adolescents. Therefore, when interpreting research results and conclusions, it is necessary to treat them differently based on the specific situation of each region.

## Conclusion and recommendations

There is a positive correlation between adolescent PAL and their levels of mental health. PA serves as a protective factor for adolescent mental health, particularly for younger boys. It is recommended that during significant natural disasters, PAL should be increased to safeguard the mental health of adolescents.

## Data Availability

The datasets used and/or analysed during the current study available from the corresponding author on reasonable request.
